# Supplementary material for: Computational Analysis of ELOVL6 Structure and Inhibition for Rational Drug Design
Source: J Chem Inf Model. 2026 Jun 5;66(12):7264–75. doi: 10.1021/acs.jcim.6c00336 (PMC13292206; doi:10.1021/acs.jcim.6c00336)
Supplement: Supplementary file 1 [file ci6c00336_si_001.pdf]

Supporting Information

Computational Analysis of ELOVL6 Structure and Inhibition for Rational Drug Design

Markel G. Ibarluzea[1,2], Rafael Ramis[1,2], Martin Fuentetaja[1,2], Francisco J Gil-Bea[6,7,10,11], Gorka Gerenu[6,9,10,11], Adolfo López De Munain[4,5,6,8,10], Jesus M. Aizpurua[12], José I. Miranda[13], Aitor Bergara[1,2,3], and Aritz Leonardo[1,2]

[1]Physics Department and EHU Quantum Center, Universidad del País Vasco-Euskal Herriko Unibertsitatea, UPV/EHU, 48080 Bilbao, Spain
[2]Donostia International Physics Center (DIPC), 20018 Donostia, Spain
[3]Centro de Física de Materiales CFM, Centro Mixto CSIC-UPV/EHU, 20018 Donostia, Spain
[4]Department of Neurology, Hospital Universitario Donostia, Osakidetza, 20014, San Sebastian, Spain
[5]Department of Neurosciences, University of the Basque Country UPV-EHU, 20014, San Sebastian, Spain
[6]Neurosciences Area, Department of Internal Medicine, Faculty of Medicine, University of Deusto, Avda. de las universidades, 48007, Bilbao, Spain
[7]Department of Health Sciences, Public University of Navarre (UPNA), Health Sciences Campus, Avda. de Barañain s/n, 31008 Pamplona, Spain
[8]Neuroscience Area, Biogipuzkoa Health Research Institute, Biodonostia Institute, 20014 San Sebastian, Spain
[9]Department of Physiology, Faculty of Medicine and Nursery, University of the Basque Country, 48940, Leioa, Spain
[10]CIBERNED, CIBER, Carlos III Institute, 28029 Madrid, Spain
[11]IKERBASQUE, Basque Foundation for Science, 48009 Bilbao, Spain
[12]Joxe Mari Korta RD Center, Department of Organic Chemistry I, University of the Basque Country, 20014 San Sebastian, Spain
[13]SGIker NMR Facility, University of Basque Country (EHU), Edificio Joxe Mari Korta Avda. de Tolosa 72 20018 San Sebastián, Spain

Contents

Molecular dynamics simulation setups

- Umbrella Sampling Simulations S3
- Mixed Solvent Molecular Dynamics S3
- Absolute Binding Free Energy (ABFE) with Membrane S3-S4
- Absolute Binding Free Energy (ABFE) in Solution S4
- Relative Binding Free Energy (RBFE) S4
- Metadynamics Simulations S4-S5

Figure S1. Lead compounds S5

Table S2-S3. Scores for IFD clusters S6-S7

Figure S2. Comparison of ELOVL6 structure prediction models S8

Figure S3. Validation of ELOVL6 structure model S9

Figure S4-S5. Evaluation of binding models S10-S11

Figure S6-S7. Absolute binding free energies for top cluster models S12

Figure S8. ROC curves of virtual screenings using constraints S13

Figure S9. Docking score distributions in virtual screenings S14

Figure S10. ELOVL6 evolutionary conservation analysis S14

Figure S11. Displacement analysis of H2 for all ELOVLs S15

Figure S12. Correlation between H2 displacement and pocket volume .. S16

Molecular Dynamics Simulation Details

Umbrella Sampling Simulations

To generate initial configurations for umbrella sampling, steered molecular dynamics (SMD) simulations were performed to explore both hypothesized substrate binding mechanisms: lateral and sequential insertion. Ten independent SMD replicas were initiated from the same starting structure. In each replica, a moving harmonic restraint was applied to the ligand to steer it along the proposed binding coordinate. The pulling rate was set to $0.01\,\mathrm{nm\,ps^{-1}}$, and a force constant of $1000\,\mathrm{kJ\,mol^{-1}\,nm^{-2}}$ was used for the moving harmonic potential. The total work performed during each trajectory was computed, and the three trajectories with the lowest total work were selected for further analysis.

From each selected trajectory, 25 structures were extracted at equidistant intervals along the reaction coordinate to define the umbrella sampling windows. A separate umbrella sampling simulation was carried out for each of the three structure sets, resulting in three umbrella sampling replicates.

All simulations were performed using GROMACS 2024.1[1] with the amber ff14SB force field for the protein[2] and GAFF2 for the substrate.[3] Systems were solvated in TIP3P water[4] with 0.15 M KCl, and long-range electrostatics were treated using Particle Mesh Ewald (PME).[5] Short-range interactions were truncated at 9 Å. Temperature was maintained at 300 K using Langevin dynamics, and pressure was kept at 1 atm using the stochastic cell rescaling barostat.[6]

Each SMD replica underwent energy minimization, followed by 0.5 ns of NVT and 0.5 ns of NPT equilibration, with positional restraints applied to protein heavy atoms. Production SMD simulations were run for 1 ns with a 2 fs timestep, applying the moving harmonic potential described above.

For each of the three selected SMD trajectories, 25 umbrella sampling windows were defined using the extracted structures. Each window was equilibrated for 10 ns under NPT conditions, followed by 75 ns of production simulation. A fixed harmonic bias with a force constant of $1000\,\mathrm{kJ\,mol^{-1}\,nm^{-2}}$ was applied in each window to restrain the system along the reaction coordinate. Simulations were conducted using the same thermostat, barostat, and cutoff settings described above, with a 2 fs timestep.

Free energy profiles along the reaction coordinate were computed using the WHAM implementation provided in GROMACS 2024.1.

Mixed Solvent Molecular Dynamics

Mixed solvent MD simulations were performed to identify potential ligand interaction hotspots on the protein surface. Five independent replicas were run, with simulation times summarized in Table S1.

All systems were built following the protocol by Bakan et al.[7] After energy minimization, a 0.6 ns annealing protocol was applied: the system was heated to 500 K and cooled to 300 K while restraining the heavy atoms of the protein. This was followed by 0.6 ns of NPT equilibration.

Simulations were run using NAMD 3.0[8] with the CHARMM36m force field.[9] The systems were solvated with TIP3P water,[4] and Cl^- ions were added for charge neutrality. Probe molecules (isopropanol, acetamide, acetate, isopropylamine, and benzene) were included at 2 M concentration.

A 2 fs timestep was used with a 12 Å cutoff for nonbonded interactions, and long-range 300 K via Langevin dynamics, and pressure was kept at 1 atm using the Langevin piston method.

Spatial distribution functions of probe molecules were analyzed using DruGUI for VMD,[10] and volumetric maps were visualized in ChimeraX.[11]

Table S1: Simulation Time for Experimental Replicas

	**Replica 1**	**Replica 2**	**Replica 3**	**Replica 4**	**Replica 5**
Simulation Time (ns)	267	274	273	268	274

Absolute Binding Free Energy (ABFE) with Membrane

ABFE simulations incorporating the presence of an explicit membrane were conducted using GROMACS 2024.1. The systems consisted of membrane-embedded protein-ligand complexes, built via CHARMM-GUI[12]

using a lipid bilayer of 90% POPC and 10% cholesterol.

The amber ff14SB, GAFF2, and LIPID21[13] force fields were used for the protein, ligand, and lipids, respectively. Solvation was performed with TIP3P water and 0.15 M KCl.

Each λ-window underwent energy minimization, followed by 0.5 ns of NPT equilibration. The production phase involved 40 ns simulation for the complex phase and 10 ns for the solvent phase. A 2 fs timestep was used with a 9 Å cutoff for short-range interactions and PME for long-range electrostatics.

Simulations were performed at 300 K using Langevin dynamics, and pressure was maintained at 1 atm using a semi-isotropic stochastic cell rescaling barostat[6] in the complex phase.

Boresch restraints[14] were applied to fix the bound ligand conformation, with atoms selected using MDRestraintsGenerator.[15] A total of 42 λ-windows were used in the complex phase and 21 in the solvent phase. Free energy differences were calculated using the BAR method[16] as implemented in GROMACS 2024.1.

Absolute Binding Free Energy (ABFE) in Solution

ABFE simulations in aqueous solution were performed using Yank v0.25.2,[17] with OpenMM 8.1.1[18] as the simulation engine. The amber ff14SB and GAFF2 force fields were used for the protein and ligands, respectively. The systems were solvated with TIP3P water and 0.15 M KCl.

Each λ-window underwent energy minimization and 1 ns equilibration, followed by 40 ns of production simulation for both the complex and solvent phases. Hamiltonian replica exchange[19] was used between windows to enhance sampling.

Simulations used a 4 fs timestep, Langevin dynamics at 300 K, and a Monte Carlo barostat at 1 atm. Short-range interactions were truncated at 9 Å, and long-range electrostatics were computed using PME.[5]

Boresch restraints were applied automatically by Yank. 42 λ-windows were used for the complex phase and 29 for the solvent phase. Final free energy estimates were obtained using Yank's internal analysis tools. Contact and hydrogen bond analyses were performed with MDTraj[20] and MDAnalysis.[21]

Relative Binding Free Energy (RBFE)

RBFE simulations were performed using OpenFE v0.25.2, with OpenMM 8.1.1 as the simulation engine. The amber ff14SB and GAFF2 force fields were used for proteins and ligands, respectively. All systems were solvated in TIP3P water with 0.15 M KCl.

Each λ-window was subjected to energy minimization and 1 ns NPT equilibration, followed by 5 ns of production simulation for both phases. For transformations showing poor convergence, simulation time was extended to 20 ns.

Simulations were carried out using Hamiltonian replica exchange across 11 λ-windows, with expansion to 25 windows where needed due to poor overlap between adjacent windows in the MBAR matrix. The temperature was kept at 300 K using Langevin dynamics, and pressure was held at 1 atm using a Monte Carlo barostat. A 4 fs timestep and 9 Å cutoff were used. PME was used for long-range electrostatics.

For the evaluation of models generated through IFD, a single replica per transformation was used, while 3 replicas per transformation were used for the validation of the best performing models. The ligand transformation network was computed based on Lomap scores,[22] and absolute binding free energies were derived from relative binding free energies using the method implemented in.[23]

Metadynamics Simulations

Enhanced sampling simulations were performed using the EDES methodology described in[24] to characterize conformational dynamics and binding pocket fluctuations in membrane-embedded systems. All systems were constructed using CHARMM-GUI, with lipid bilayers composed of 90% POPC and 10% cholesterol, consistent with the membrane setups used in the ABFE simulations described above.

Simulations were carried out using GROMACS 2024.4 patched with the open-source, community-developed PLUMED library,[25] version 2.9.2.[26] The same force field as those employed in the ABFE membrane simulations were used throughout, namely the amber ff14SB, GAFF2, and LIPID2113 force fields were used for the protein, ligand, and lipids, respectively. Systems were solvated in TIP3P water with 0.15 M KCl,

and long-range electrostatics were treated using the Particle Mesh Ewald (PME) method with a 9 cutoff for short-range interactions. Simulations were performed at 300 K using Langevin dynamics, with pressure maintained at 1 atm using a semi-isotropic stochastic cell rescaling barostat. A 2 fs timestep was employed.

Prior to enhanced sampling, each system was equilibrated and subsequently simulated for 1 ns to characterize the natural fluctuations of the chosen collective variables (CVs). The standard deviation of each CV was computed from these trajectories and used to define the Gaussian width (σ) for the metadynamics bias, set as $0.375 \times \sigma$.

Enhanced sampling simulations were performed using bias-exchange well-tempered metadynamics, with one replica for each of the four CVs employed. The collective variables along which dynamics were enhanced were the radius of gyration of the binding pocket, and the coordination numbers along the planes orthogonal to the principal axes of inertia of the atoms in the binding pocket. Gaussian hills with a height of $0.6\,\mathrm{kcal\,mol^{-1}}$ were deposited every 1000 steps, with a bias factor of 10. Each of the replicas were simulated for a total of 500 ns, resulting in an aggregate simulation time of 2 µs. Exchanges between replicas with different bias potentials were attempted every 10000 steps.

Binding pocket volume analysis along the trajectories was carried out using fpocket.[27]

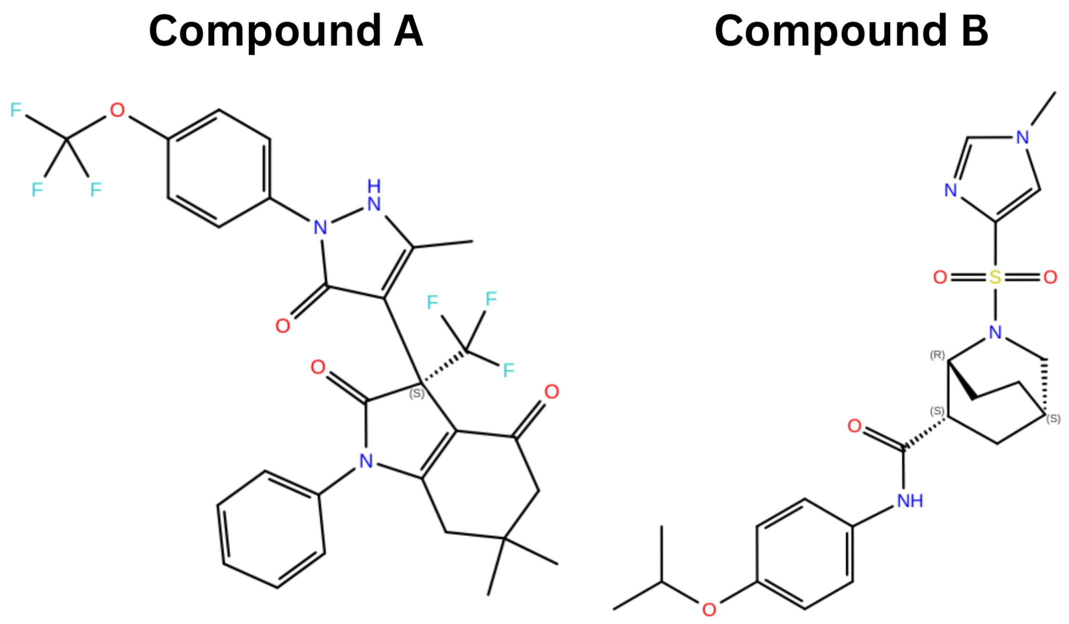

Figure S1: (A) Lead compounds selected from two congenertic series of ELOVL6 inhibitors.

Table S2: Docking scores, induced fit docking scores and CompScore from binding pose metadynamics simulations, computed following,[28] for compound A. Data for each of the 5 best selected clusters obtained from the IFD simulations of all 5 AlphaFold models is shown.

**Model**	**Cluster**	**CompScore**	**IFDScore**	**Docking Score**
Model 1	Cluster 1	-3.48	-11376.91	-11.96
	Cluster 2	1.45	-11330.38	-12.38
	Cluster 3	-1.44	-11285.65	-8.90
	Cluster 4	-2.35	-11338.03	-9.89
	Cluster 5	1.72	-11313.85	-8.73
Model 2	Cluster 1	-2.17	-11319.64	-11.09
	Cluster 2	-1.21	-11300.67	-8.08
	Cluster 3	-0.78	-11313.93	-10.49
	Cluster 4	1.33	-11331.27	-11.89
	Cluster 5	-1.35	-11313.67	-10.04
Model 3	Cluster 1	-1.05	-11345.90	-10.00
	Cluster 2	0.21	-11362.96	-11.21
	Cluster 3	-0.67	-11312.65	-9.02
	Cluster 4	-0.08	-11408.97	-11.32
	Cluster 5	-0.71	-11306.65	-10.22
Model 4	Cluster 1	-0.36	-11391.44	-9.11
	Cluster 2	0.43	-11399.33	-10.15
	Cluster 3	1.10	-11357.53	-10.41
	Cluster 4	-0.59	-11386.28	-9.15
	Cluster 5	-1.88	-11387.47	-10.60
Model 5	Cluster 1	-0.07	-11303.85	-10.32
	Cluster 2	-1.13	-11372.21	-9.93
	Cluster 3	-1.20	-11360.27	-9.37
	Cluster 4	-0.20	-11304.95	-11.26
	Cluster 5	1.63	-11337.46	-11.08

Table S3: Docking scores, induced fit docking scores and CompScore from binding pose metadynamics simulations, computed following,[28] for compound B. Data for each of the 5 best selected clusters obtained from the IFD simulations of all 5 AlphaFold models is shown.

Model	Cluster	CompScore	IFDScore	Docking Score
Model 1	Cluster 1	-1.03	-11384.68	-8.45
	Cluster 2	1.54	-11414.41	-8.70
	Cluster 3	-0.15	-11417.89	-9.11
	Cluster 4	-0.53	-11373.11	-9.95
	Cluster 5	0.02	-11381.47	-10.36
Model 2	Cluster 1	-0.93	-11326.63	-9.00
	Cluster 2	1.28	-11323.93	-7.51
	Cluster 3	1.28	-11306.00	-7.16
	Cluster 4	0.43	-11290.96	-7.35
	Cluster 5	-1.83	-11357.01	-9.03
Model 3	Cluster 1	-0.08	-11341.53	-7.40
	Cluster 2	-0.78	-11368.36	-9.21
	Cluster 3	1.68	-11377.64	-9.22
	Cluster 4	-2.23	-11384.47	-9.20
	Cluster 5	0.25	-11338.72	-8.12
Model 4	Cluster 1	2.44	-11386.66	-9.07
	Cluster 2	-0.68	-11370.32	-9.55
	Cluster 3	-0.21	-11389.09	-7.19
	Cluster 4	1.93	-11403.79	-8.47
	Cluster 5	-1.88	-11302.75	-6.86
Model 5	Cluster 1	0.06	-11396.06	-9.53
	Cluster 2	-2.99	-11386.91	-7.24
	Cluster 3	0.45	-11416.65	-8.00
	Cluster 4	-1.22	-11403.45	-7.10
	Cluster 5	2.06	-11391.49	-8.21

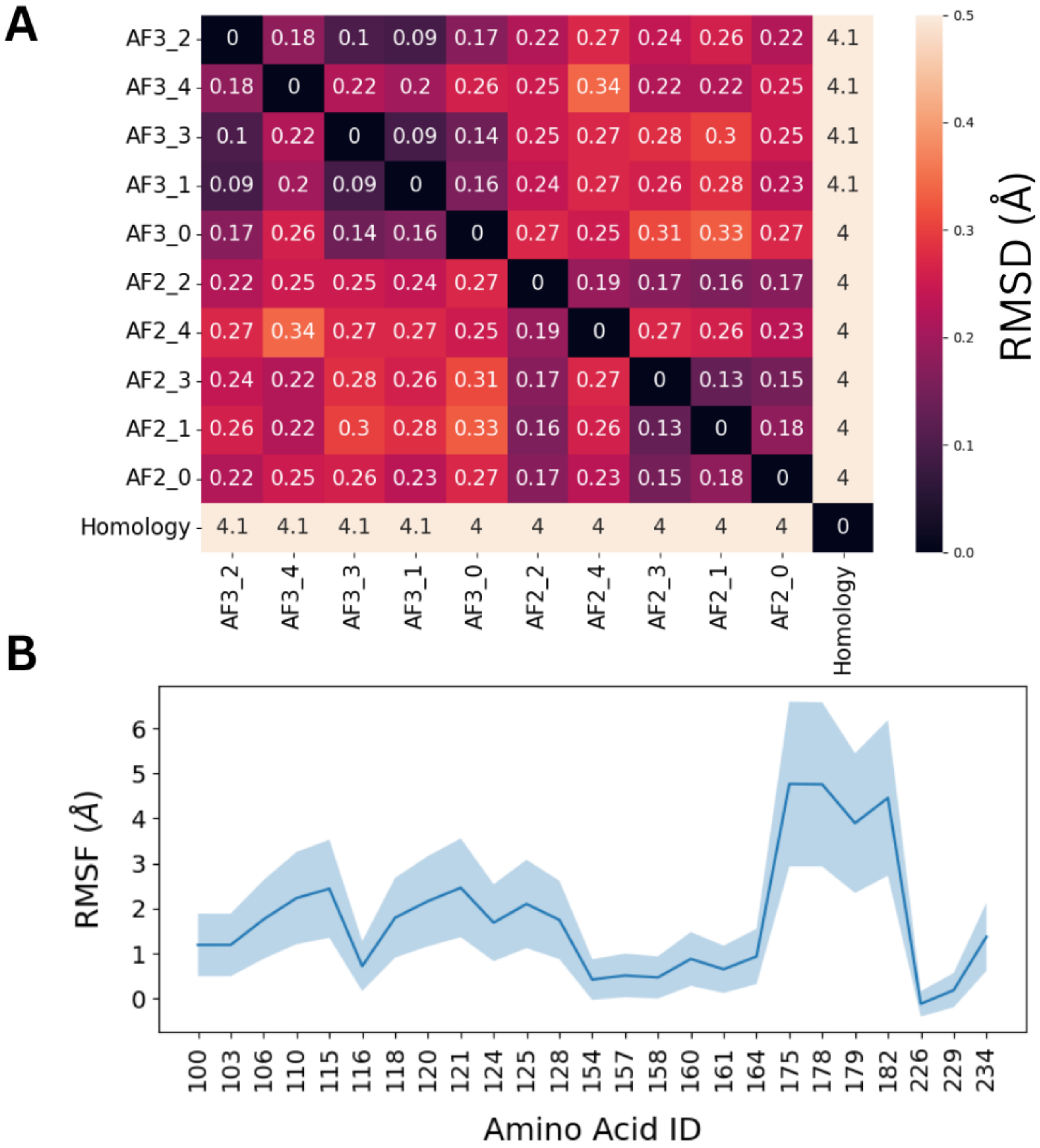

Figure S2: (A) Heatmap of average backbone RMSDs between ELOVL6 models generated through AlphaFold2, AlphaFold3, and a homology model of ELOVL6 constructed using ELOVL7s crystal structure as template. (B) RMSF of binding site amino acids between the homology model and AlphaFold models.

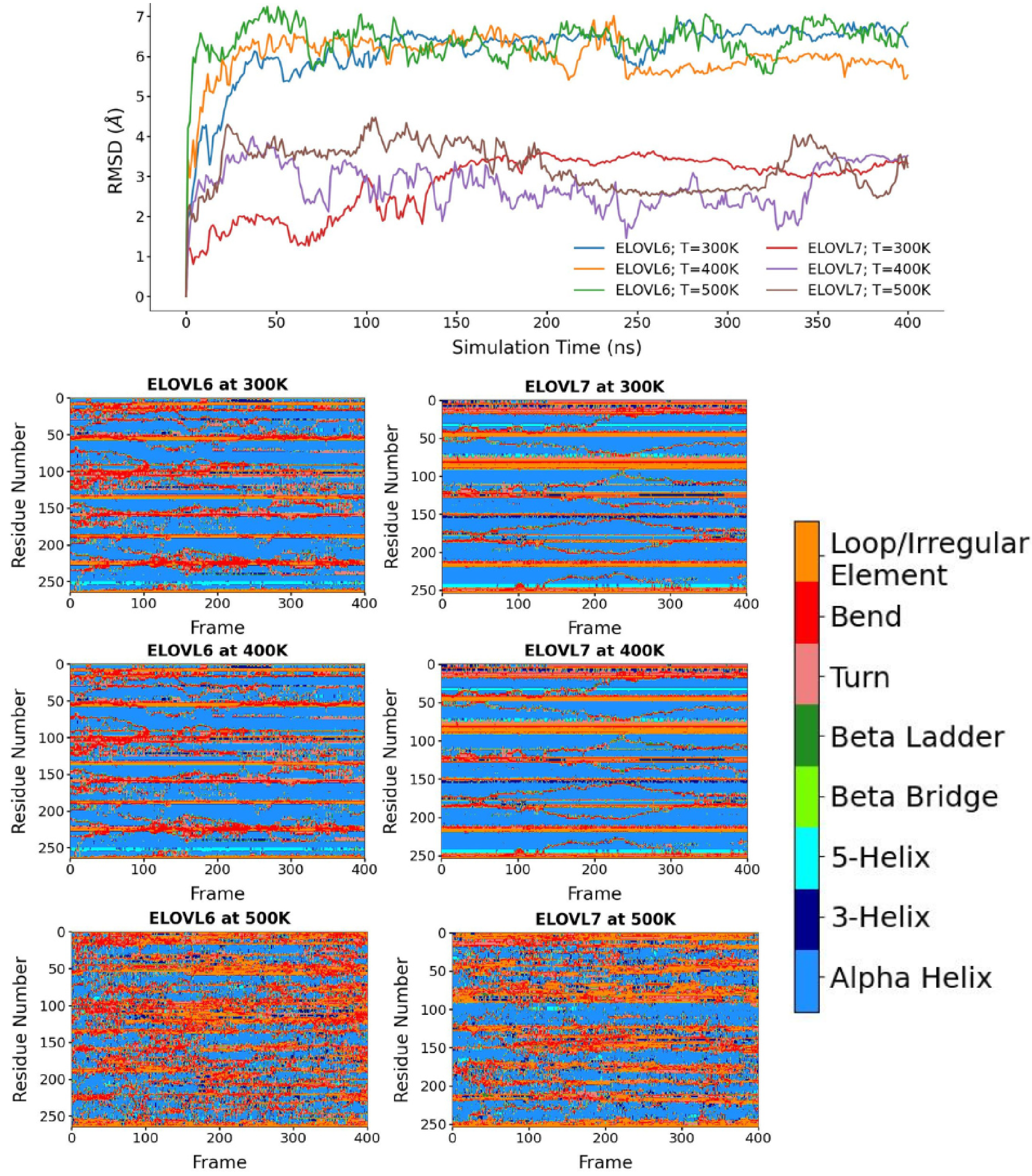

Figure S3: (A) Backbone RMSD values of the best AlphaFold model for ELOVL6 and the crystal structure of ELOVL7 (PDBID 6Y7F) obtained from independent simulations of both proteins at temperatures of 300K, 400K and 500K. (B) Time evolution of the secondary structure of ELOVL6 structural model and ELOVL7 crystal structure at temperatures of 300K, 400K and 500K.

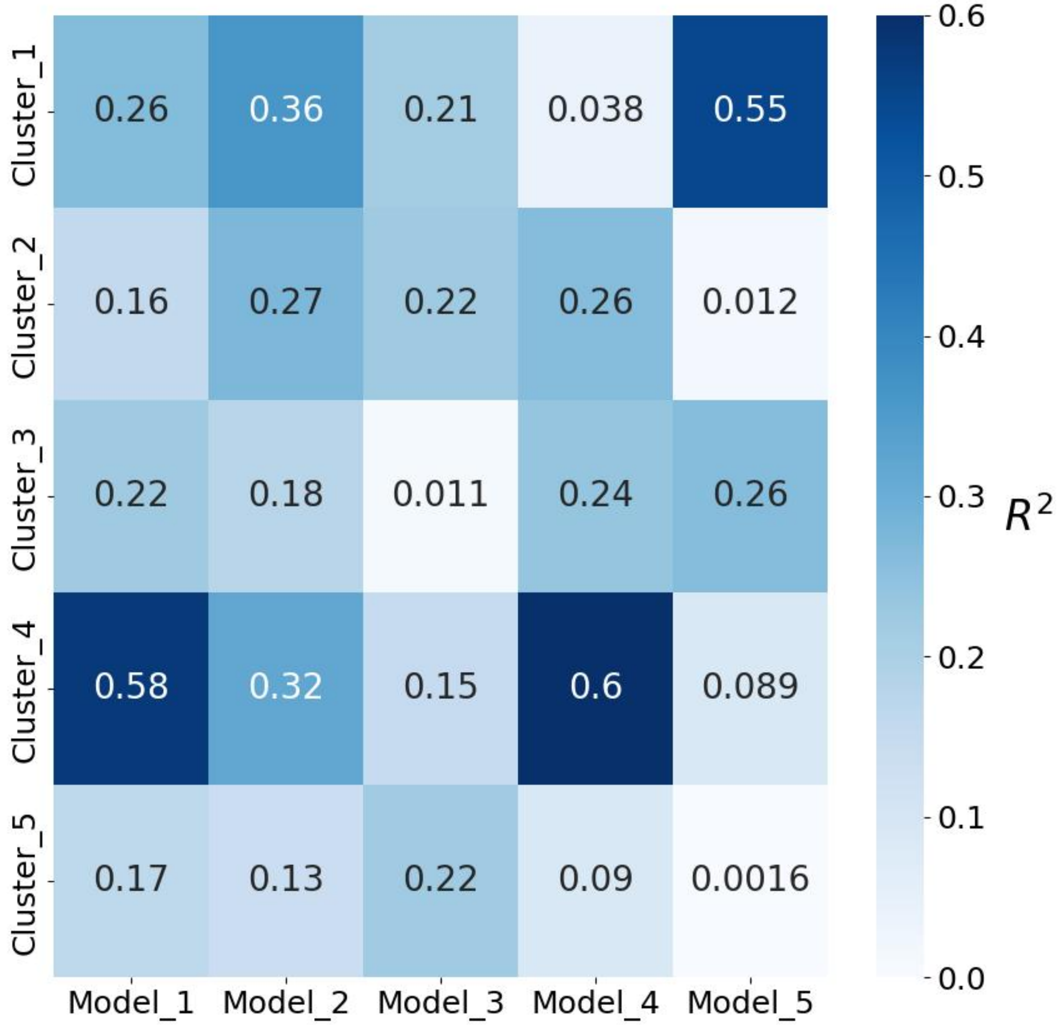

Figure S4: Heatmap representing the performance of all bound conformations of compound A, evaluated through the correlation between experimental binding free energy differences and binding free energy differences calculated from RBFE simulations of a subset of compound A's derivatives.

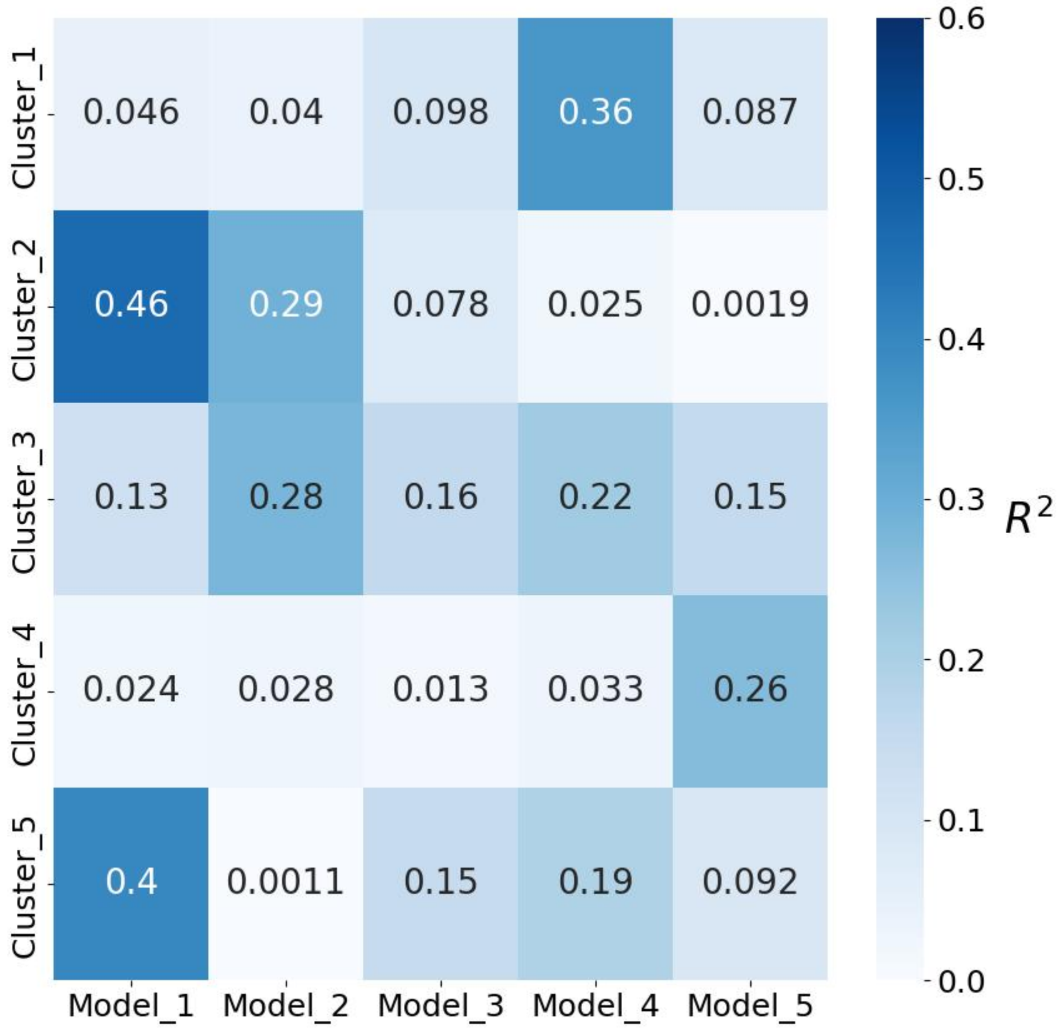

Figure S5: Heatmap representing the performance of all bound conformations of compound B, evaluated through the correlation between experimental binding free energy differences and binding free energy differences calculated from RBFE simulations of a subset of compound B's derivatives.

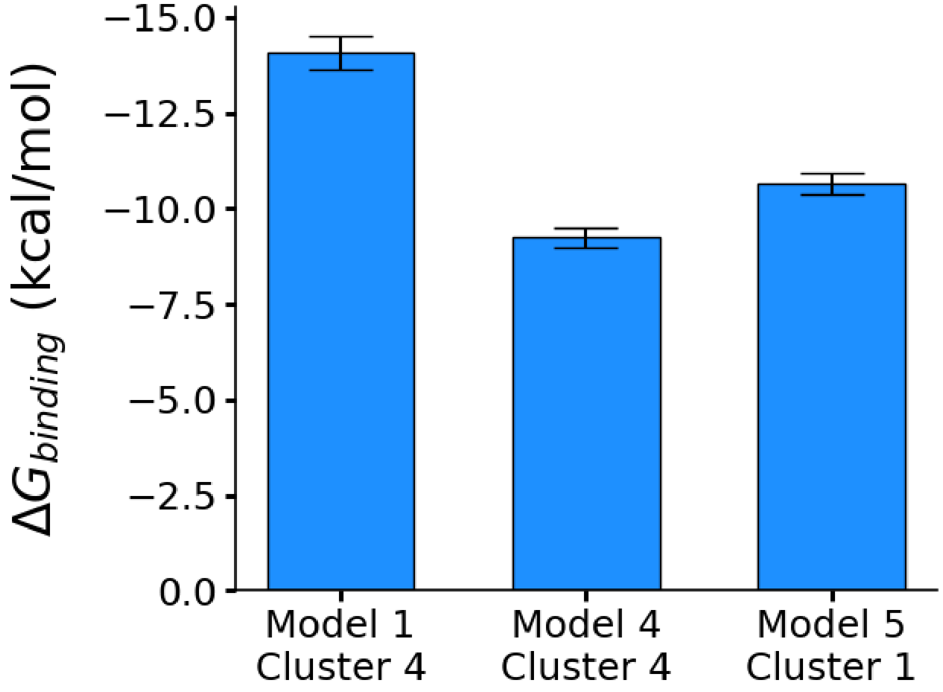

Figure S6: Absolute binding free energies for the 3 best performing IFD poses of compound A, based on the correlation values from figure 3. Poses with coefficients of determination above 0.4 were simulated.

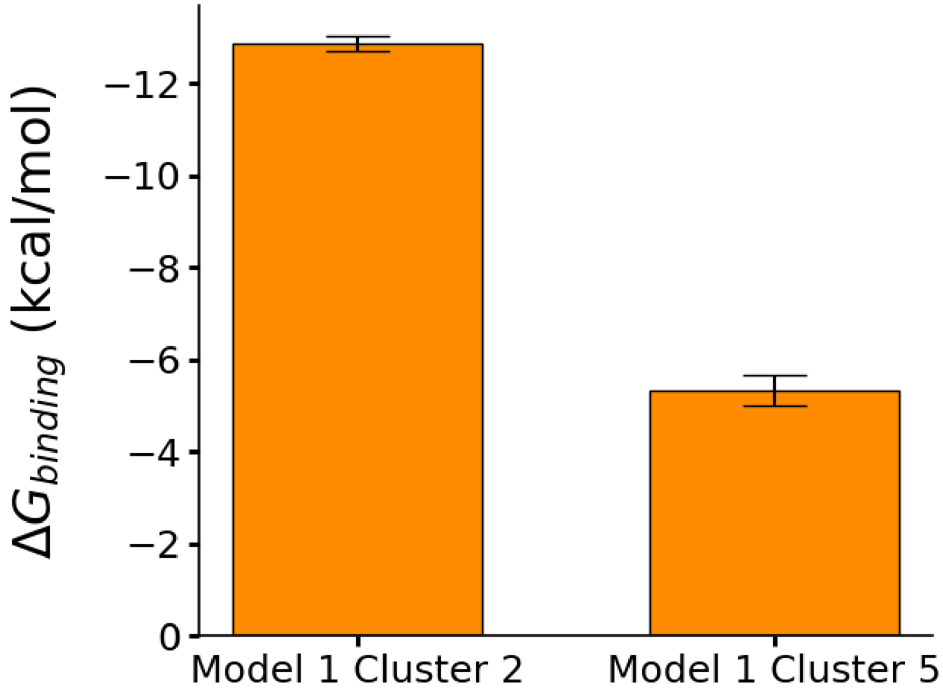

Figure S7: Absolute binding free energies for the 2 best performing IFD poses of compound B, based on the correlation values from figure 4. Poses with coefficients of determination above 0.4 were simulated.

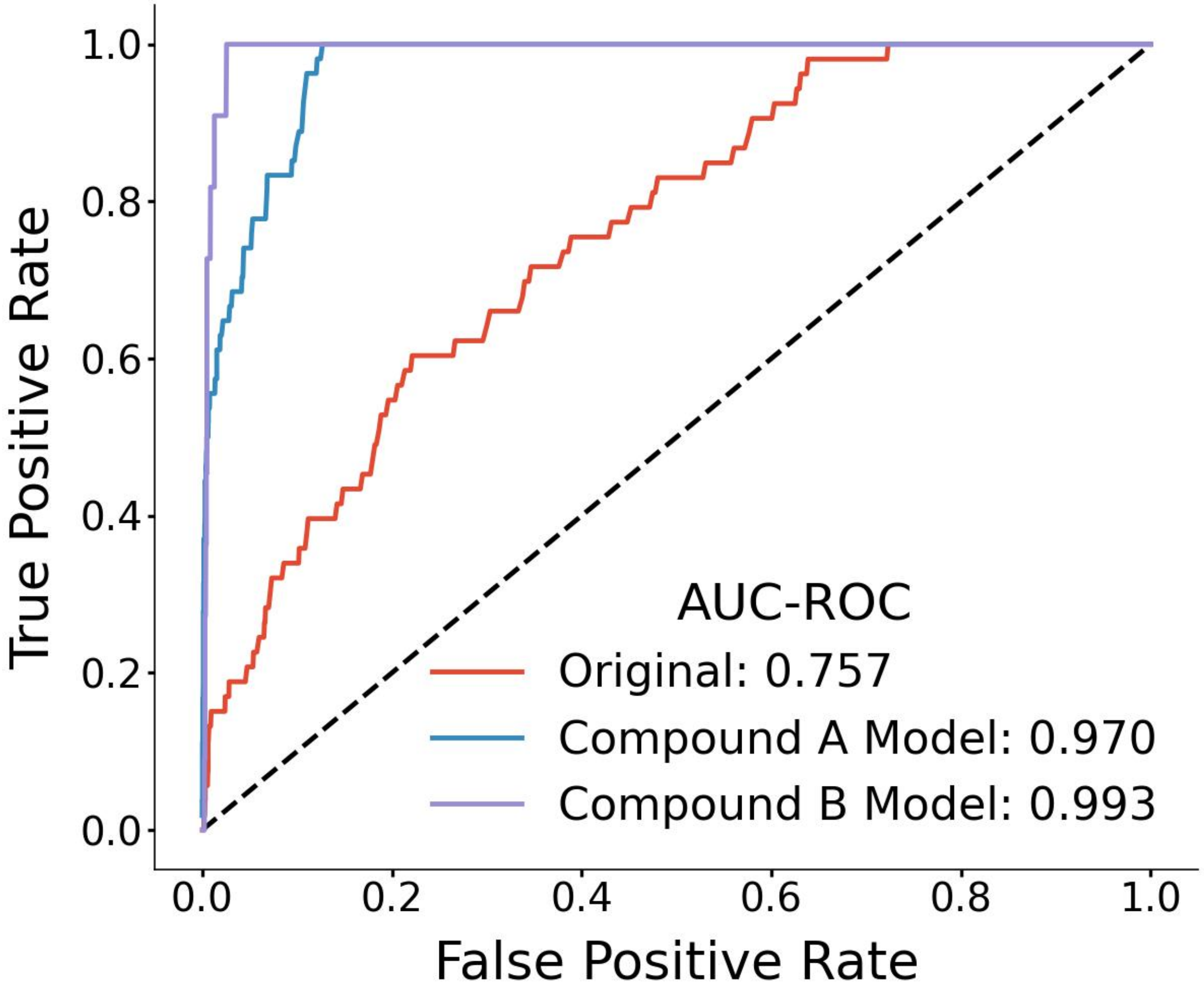

Figure S8: ROC curves for the retrospective virtual screenings using the original AlphaFold model, and the optimal models for ligand series 1 and 2, with constraints on the allowed docking poses to require hydrogen bond formation with residues TYR181 and/or GLN202.

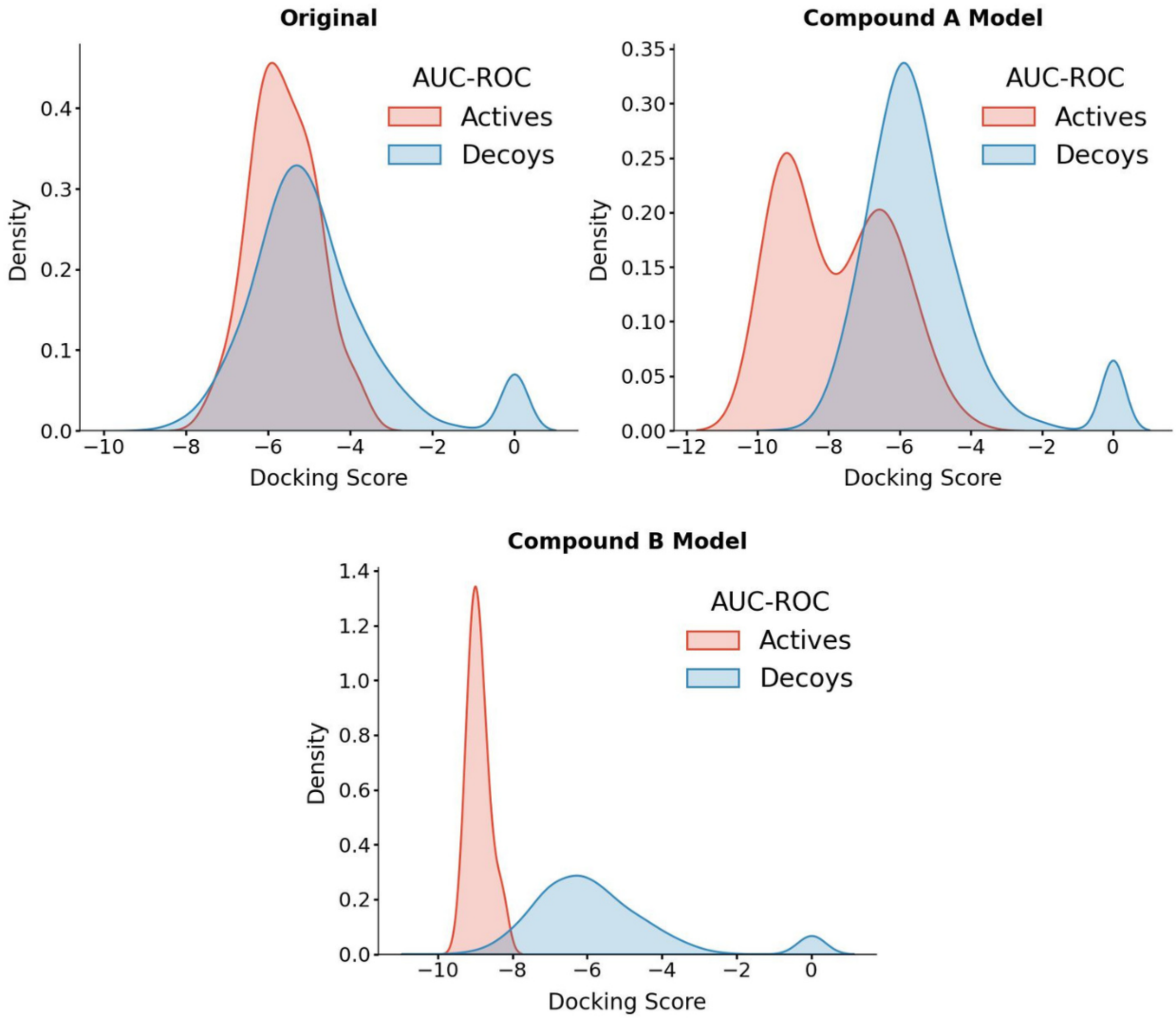

Figure S9: Comparison between the the docking score distributions of known active molecules and decoy molecules for (A) the original AlphaFold model, (B) optimal model for compound A and (C) optimal model for compound B, showing a clear distributional shift towards better docking scores for known actives compared to decoys.

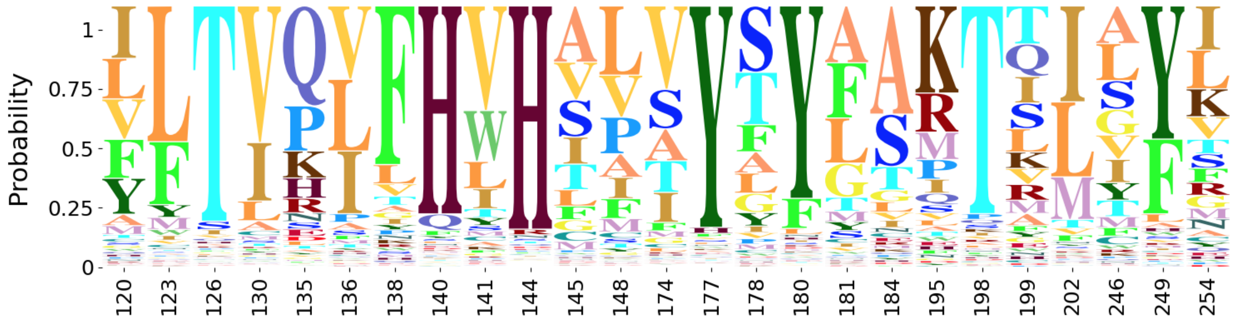

Figure S10: LogO representation of evolutionary conservation analysis of binding site residues for ELOVL6.

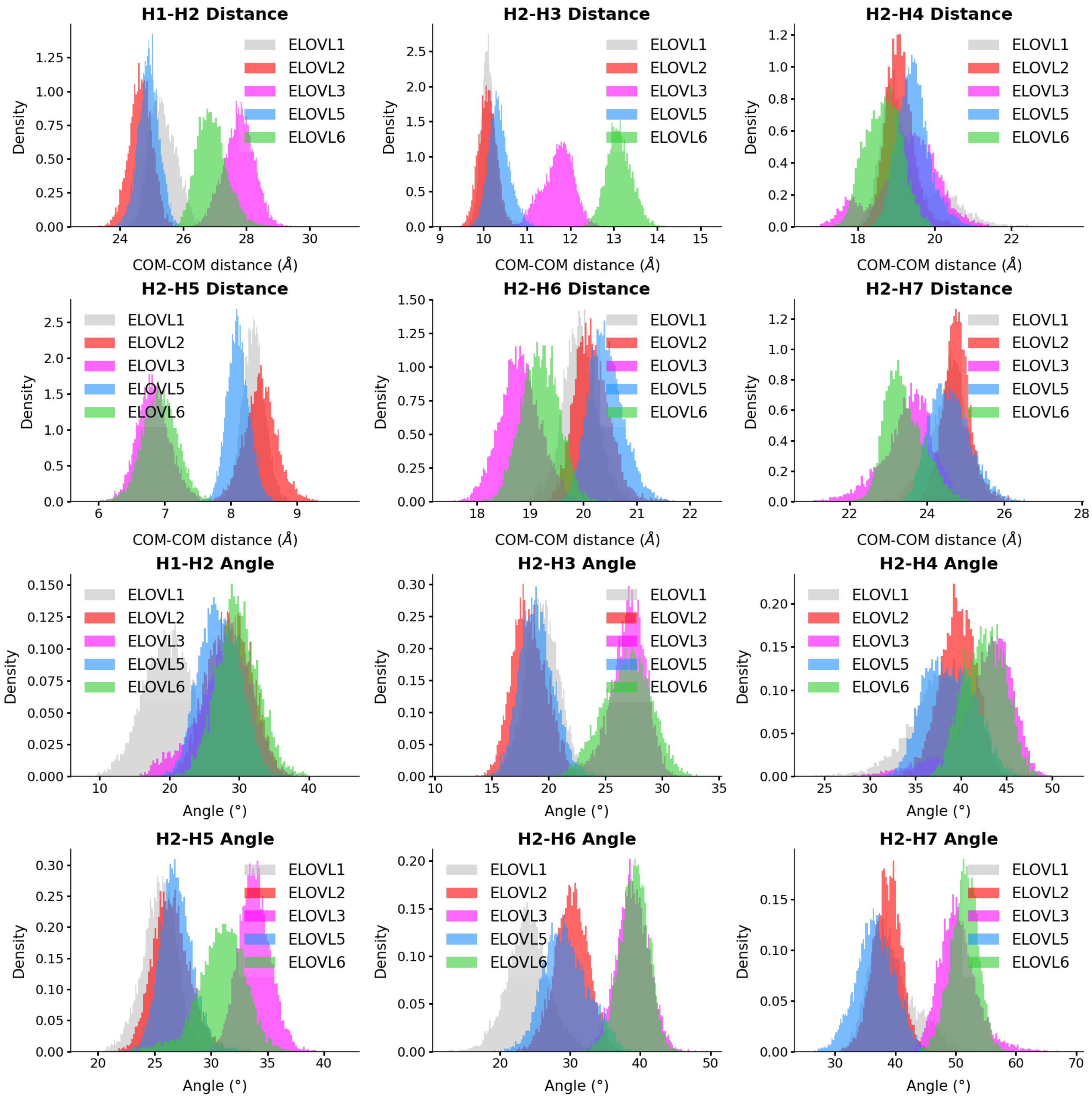

Figure S11: Reweighted histograms of the pairwise distances and angles between helix H2 and the rest of the helices that make up the ELOVL family proteins. Various pairwise measures show a clear separation between the behaviors of ELOVL6 and ELOVL3 compared to those of ELOVL1, ELOVL2 and ELOVL5.

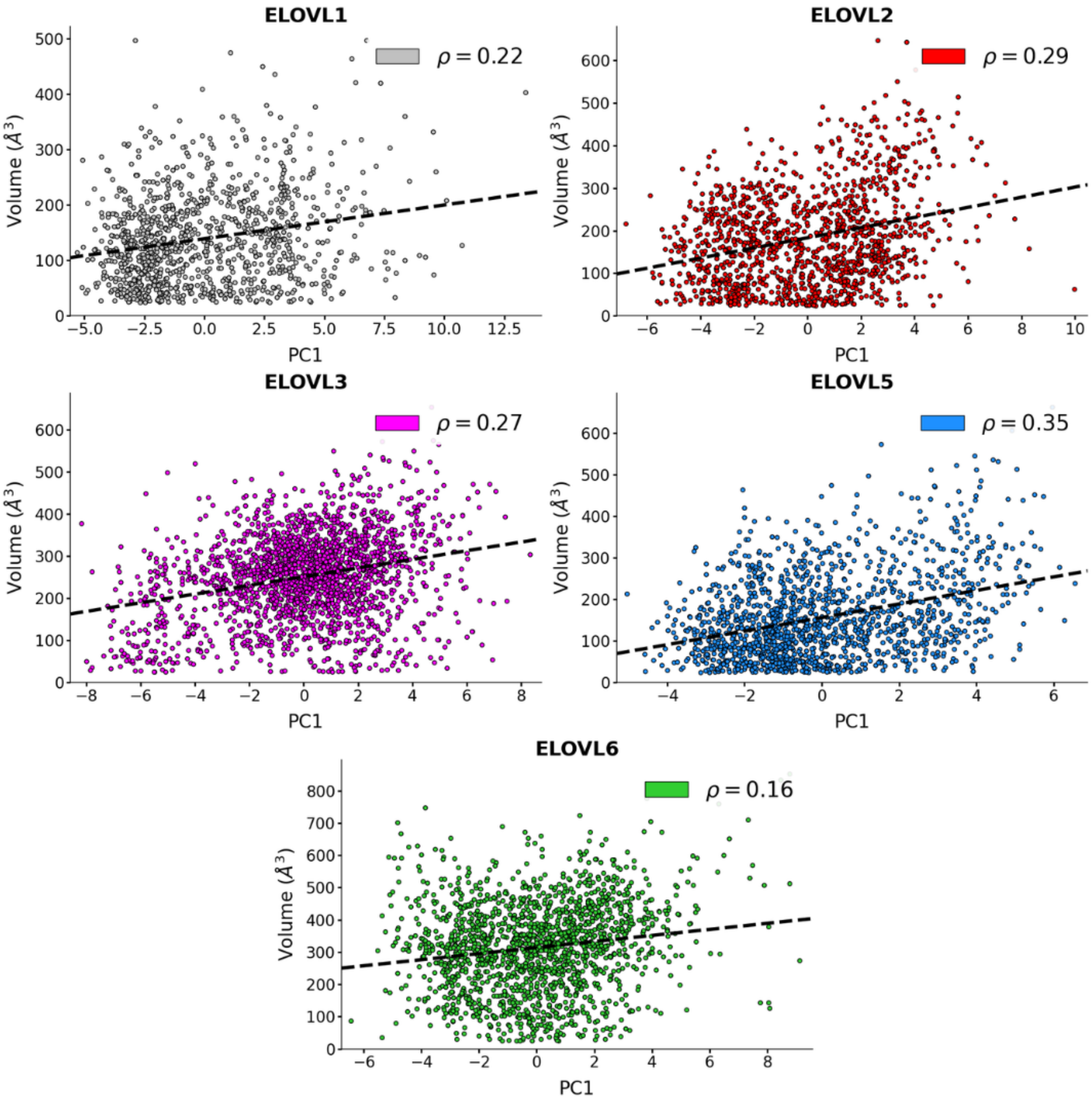

Figure S12: Relationship between the displacement of the H2 helix and the volume of the binding pocket. The various pairwise displacement measures (distances and angles) of helix H2 with respect to other helices are projected onto the first principal component after processing with PCA. The correlation ρ between H2 displacements first principal component and the volume of the binding pocket of each ELOVL member is measured using Pearson correlation.

References

[1] Abraham, M. J.; Murtola, T.; Schulz, R.; Páll, S.; Smith, J. C.; Hess, B.; Lindahl, E. GROMACS: High performance molecular simulations through multi-level parallelism from laptops to supercomputers. *SoftwareX* **2015**, *1*, 19–25.

[2] Maier, J. A.; Martinez, C.; Kasavajhala, K.; Wickstrom, L.; Hauser, K. E.; Simmerling, C. ff14SB: improving the accuracy of protein side chain and backbone parameters from ff99SB. *Journal of chemical theory and computation* **2015**, *11*, 3696–3713.

[3] Wang, J.; Wolf, R. M.; Caldwell, J. W.; Kollman, P. A.; Case, D. A. Development and testing of a general amber force field. *Journal of computational chemistry* **2004**, *25*, 1157–1174.

[4] Jorgensen, W. L.; Chandrasekhar, J.; Madura, J. D.; Impey, R. W.; Klein, M. L. Comparison of simple potential functions for simulating liquid water. *The Journal of chemical physics* **1983**, *79*, 926–935.

[5] Darden, T.; York, D.; Pedersen, L.; others Particle mesh Ewald: An N log (N) method for Ewald sums in large systems. *Journal of chemical physics* **1993**, *98*, 10089–10089.

[6] Bussi, G.; Zykova-Timan, T.; Parrinello, M. Isothermal-isobaric molecular dynamics using stochastic velocity rescaling. *The Journal of chemical physics* **2009**, *130*.

[7] Bakan, A.; Nevins, N.; Lakdawala, A. S.; Bahar, I. Druggability assessment of allosteric proteins by dynamics simulations in the presence of probe molecules. *Journal of chemical theory and computation* **2012**, *8*, 2435–2447.

[8] Phillips, J. C.; Hardy, D. J.; Maia, J. D.; Stone, J. E.; Ribeiro, J. V.; Bernardi, R. C.; Buch, R.; Fiorin, G.; Hénin, J.; Jiang, W.; others Scalable molecular dynamics on CPU and GPU architectures with NAMD. *The Journal of chemical physics* **2020**, *153*.

[9] Huang, J.; Rauscher, S.; Nawrocki, G.; Ran, T.; Feig, M.; De Groot, B. L.; Grubmüller, H.; MacKerell Jr, A. D. CHARMM36m: an improved force field for folded and intrinsically disordered proteins. *Nature methods* **2017**, *14*, 71–73.

[10] Humphrey, W.; Dalke, A.; Schulten, K. VMD: visual molecular dynamics. *Journal of molecular graphics* **1996**, *14*, 33–38.

[11] Pettersen, E. F.; Goddard, T. D.; Huang, C. C.; Meng, E. C.; Couch, G. S.; Croll, T. I.; Morris, J. H.; Ferrin, T. E. UCSF ChimeraX: Structure visualization for researchers, educators, and developers. *Protein science* **2021**, *30*, 70–82.

[12] Jo, S.; Kim, T.; Iyer, V. G.; Im, W. CHARMM-GUI: a web-based graphical user interface for CHARMM. *Journal of computational chemistry* **2008**, *29*, 1859–1865.

[13] Dickson, C. J.; Walker, R. C.; Gould, I. R. Lipid21: complex lipid membrane simulations with AMBER. *Journal of chemical theory and computation* **2022**, *18*, 1726–1736.

[14] Boresch, S.; Tettinger, F.; Leitgeb, M.; Karplus, M. Absolute binding free energies: a quantitative approach for their calculation. *The Journal of Physical Chemistry B* **2003**, *107*, 9535–9551.

[15] Alibay, I.; Magarkar, A.; Seeliger, D.; Biggin, P. C. Evaluating the use of absolute binding free energy in the fragment optimisation process. *Communications Chemistry* **2022**, *5*, 105.

[16] Bennett, C. H. Efficient estimation of free energy differences from Monte Carlo data. *Journal of Computational Physics* **1976**, *22*, 245–268.

[17] Rizzi, A.; Grinaway, P.; Parton, D.; Shirts, M. R.; Wang, K.; Eastman, P.; Friedrichs, M.; Pande, V. S.; Branson, K.; Mobley, D.; others YANK: a GPU-accelerated platform for alchemical free energy calculations. *can be found under getyank. org* **2020**,

[18] Eastman, P.; Swails, J.; Chodera, J. D.; McGibbon, R. T.; Zhao, Y.; Beauchamp, K. A.; Wang, L.-P.; Simmonett, A. C.; Harrigan, M. P.; Stern, C. D.; others OpenMM 7: Rapid development of high performance algorithms for molecular dynamics. *PLoS computational biology* **2017**, *13*, e1005659.

[19] Sugita, Y.; Okamoto, Y. Replica-exchange molecular dynamics method for protein folding. *Chemical physics letters* **1999**, *314*, 141–151.

[20] McGibbon, R. T.; Beauchamp, K. A.; Harrigan, M. P.; Klein, C.; Swails, J. M.; Hernández, C. X.; Schwantes, C. R.; Wang, L.-P.; Lane, T. J.; Pande, V. S. MDTraj: a modern open library for the analysis of molecular dynamics trajectories. *Biophysical journal* **2015**, *109*, 1528–1532.

[21] Michaud-Agrawal, N.; Denning, E. J.; Woolf, T. B.; Beckstein, O. MDAnalysis: a toolkit for the analysis of molecular dynamics simulations. *Journal of computational chemistry* **2011**, *32*, 2319–2327.

[22] Liu, S.; Wu, Y.; Lin, T.; Abel, R.; Redmann, J. P.; Summa, C. M.; Jaber, V. R.; Lim, N. M.; Mobley, D. L. Lead optimization mapper: automating free energy calculations for lead optimization. *Journal of computer-aided molecular design* **2013**, *27*, 755–770.

[23] Li, Y.; Liu, R.; Liu, J.; Luo, H.; Wu, C.; Li, Z. An open source graph-based weighted cycle closure method for relative binding free energy calculations. *Journal of Chemical Information and Modeling* **2022**, *63*, 561–570.

[24] Basciu, A.; Malloci, G.; Pietrucci, F.; Bonvin, A. M.; Vargiu, A. V. Holo-like and druggable protein conformations from enhanced sampling of binding pocket volume and shape. *Journal of Chemical Information and Modeling* **2019**, *59*, 1515–1528.

[25] Promoting transparency and reproducibility in enhanced molecular simulations. *Nature methods* **2019**, *16*, 670–673.

[26] Tribello, G. A.; Bonomi, M.; Branduardi, D.; Camilloni, C.; Bussi, G. PLUMED 2: New feathers for an old bird. *Computer physics communications* **2014**, *185*, 604–613.

[27] Le Guilloux, V.; Schmidtke, P.; Tuffery, P. Fpocket: an open source platform for ligand pocket detection. *BMC bioinformatics* **2009**, *10*, 168.

[28] Fusani, L.; Palmer, D. S.; Somers, D. O.; Wall, I. D. Exploring ligand stability in protein crystal structures using binding pose metadynamics. *Journal of Chemical Information and Modeling* **2020**, *60*, 1528–1539.
